# Multidimensional Classification of Insulin Resistance (MCIR) Focused on Therapeutic Targets and Interventions—A Comprehensive Review

**DOI:** 10.3390/ijms27135876

**Published:** 2026-06-30

**Authors:** Dominik Nowakowski, Adrianna Zańko, Michał Pawłowski, Robert Milewski

**Affiliations:** Department of Biostatistics and Medical Informatics, Medical University of Bialystok, 15-295 Bialystok, Poland; dominik.nowakowski@umb.edu.pl (D.N.); adrianna.zanko@umb.edu.pl (A.Z.); michal.pawlowski@umb.edu.pl (M.P.)

**Keywords:** insulin resistance, molecular pathways, classification, IR types, metabolic disorders, phenotype

## Abstract

Insulin resistance (IR) is a common medical condition that plays a crucial role in the development of numerous diseases. Even though its overall mechanism is fairly simple and involves an insufficient response of tissues to insulin signaling resulting in increased hepatic glucose production, the condition’s presentation and etiology are complex enough to warrant a broad review and re-classification. The aim of this study was to review the current scientific consensus concerning IR and its various manifestations and, based on the conclusions, create a comprehensive classification that would be focused on therapeutic aims and interventions catered to the various types of the condition. Using the available literature data, the existing approaches have been critically reviewed and collated into a novel classification of types of insulin resistance, named the Multidimensional Classification of Insulin Resistance (MCIR). As the review and the MCIR based on it provide useful insight into the biological phenomena involved in IR and clarify which therapeutic approaches and interventions are indicated for different types of IR, the results of this study should prove useful in clinical contexts. Further research into the condition is warranted because, despite its high prevalence, IR remains characterized by substantial empirical uncertainty, with various approaches and concepts often developing independently with insufficient overlap and consistency.

## 1. Introduction

Resistance to insulin-stimulated glucose uptake, i.e., insulin resistance (IR), is a common phenomenon that plays a central role in the development and clinical progression of several major human diseases such as type 2 diabetes, hypertension, or coronary artery disease [1]. In healthy individuals, pancreatic β-cells respond to increased blood glucose levels by secreting appropriate amounts of insulin, which inhibits glucose production in the liver [2]. In individuals with IR, despite increased insulin production, tissues respond as if the level of insulin were insufficient, e.g., by producing more glucose in the liver [3], and/or they do not uptake and utilize glucose sufficiently. In addition to regulating blood glucose levels, insulin also plays a crucial role in lipid level control and regulation. Increased hepatic lipogenesis is observed in IR, which can ultimately lead to nonalcoholic fatty liver disease, among other conditions [4]. Lipid accumulation in non-adipose tissues is associated with β-cell hypertrophy, which exacerbates IR, thus creating a cycle of metabolic abnormalities [5]. Insulin resistance is not considered a disease, but rather a temporary medical condition that impairs the body’s functioning. However, untreated IR can lead to numerous metabolic and cardiovascular disorders—directly to type 2 diabetes, and indirectly to, for example, cardiovascular events, metabolic syndrome, obesity, and infertility [6,7]. Currently, insulin resistance is understood as a dynamic phenomenon that depends on the metabolic context.

The concept of insulin resistance was introduced in 1934 by Himsworth [8], who examined glucose tolerance after carbohydrate consumption and the metabolic response to exogenous insulin administration in a group of diabetic patients. His aim was to establish whether disturbances of carbohydrate metabolism result from insulin deficiency or from impaired tissue responsiveness to insulin. He demonstrated that both mechanisms may occur in different patients; i.e., the presence of insulin does not guarantee its metabolic activity. In 1988, Reaven [1] showed that IR can exist without the presence of overt diabetes and characterized it as the condition whose role is crucial in the development of metabolic syndrome. Hence, resistance to insulin resulting from impaired tissue response became recognized as a clinically relevant concept.

Randle et al. [9] suggested that an excess of fatty acids may impair the action of insulin in muscles. This hypothesis was termed the glucose-fatty acid cycle (or Randle cycle) hypothesis. The concept of insulin resistance was further refined in 1988 when it was proposed as a central pathogenic mechanism underlying metabolic syndrome [1]. The same year, DeFronzo [10] proposed the triumvirate, which was based on the idea that the liver, muscles, and the pancreas are the three key organs involved in the development of type 2 diabetes, which laid the groundwork for the concept of organ-specific (or tissue-specific) insulin resistance.

Due to the potential risk of developing lifestyle-related diseases, quantifying insulin sensitivity or resistance in humans is of great importance for epidemiological studies and clinical practice. Currently, several diagnostic methods dedicated to IR exist, ranging from direct measures, such as the hyperinsulinemic–euglycemic glucose clamp (i.e., the clamp) or the insulin suppression test, to indirect surrogate measures such as the Matsuda or HOMA-IR indices. The clamp—considered the gold standard in assessing insulin resistance—directly measures insulin-dependent glucose utilization under steady-state conditions. However, this method has its notable limitations; i.e., it is time-consuming, labor-intensive, and has very limited availability in clinical practice. Therefore, indirect methods, such as calculating the HOMA-IR or Matsuda indices, constitute a clinically simpler—if less accurate—approach. In the case of the HOMA-IR index, only fasting glucose and insulin concentrations are used to assess IR. It is calculated using the following formula: fasting insulin (μIU/mL) × fasting glucose (mmol/L)/22.5. The Matsuda index, on the other hand, is calculated based on the oral glucose tolerance test [11], i.e., 10,000/√ ([fasting glucose (mmol/L) × fasting insulin (pmol/L)] × [mean glucose (mmol/L) × mean insulin (pmol/L) measured during the OGTT]) [12]. Despite the variety of tests that assess insulin sensitivity and resistance, the lack of a precise definition of the key term and the lack of consistency in its assessment result in IR being a very complex clinical problem.

The classification of IR varies depending on the approach. This stems from the fact that insulin resistance itself is a condition that operates on multiple levels and affects various organs and processes. For instance, a disruption of the balance between satiety and hunger signals can be observed in the hypothalamus, which may lead to excessive calorie consumption. Theoretically, in healthy individuals, this excess energy should be stored in adipose tissue, but under the influence of inflammation—often co-existing with IR—this ability is impaired. Consequently, increased lipid release into the bloodstream is accompanied by deposition of fat molecules in the liver or skeletal muscle. This phenomenon is called lipotoxicity, which exacerbates IR [13]. Additionally, the classification of insulin resistance into types A and B is sometimes proposed, depending on whether the cause of insulin resistance is the presence of genetic abnormalities or the presence of antibodies against the insulin receptor [14]. Difficulties in understanding the nature of IR also arise in the context of diagnostics. Different methods for measuring insulin resistance assess distinct components of the condition. Of the most common ones, the HOMA-IR index primarily estimates hepatic IR in the fasting state, whereas the clamp mainly measures peripheral (especially skeletal muscle) insulin sensitivity. The Matsuda index, on the other hand, primarily estimates hepatic and peripheral insulin sensitivity [11]. Due to the aforementioned discrepancies and ambiguities, introducing a novel comprehensive classification that would take into account all the facts about IR that have been established so far—one that would be adapted to current clinical needs and provide a detailed picture of the condition—is warranted.

Hence, the aim of this article is to provide an organized overview of the types of insulin resistance identified in the literature in the form of a structured and clustered Multidimensional Classification of Insulin Resistance (MCIR) focused on therapeutic aims. Current evidence indicates that insulin resistance is a heterogeneous phenomenon involving various tissues, molecular mechanisms, and clinical manifestations. Therefore, the proposed classification aims to provide a comprehensive framework that may facilitate future research, improve communication between researchers and clinicians, enable more precise diagnosis, and help identify mechanism-specific therapeutic targets. The framework is based on an extensive review of the literature data along with an in-depth analysis of the underlying mechanisms of action of IR. Additionally, the identification of potential therapeutic targets that should be approached in the treatment of insulin resistance is attempted.

## 2. Materials and Methods

The study was conducted as a literature review covering publications available up to 2025 retrieved from the following databases: PubMed/Medline, EMBASE (Elsevier), Scopus, Web of Science, and Google Scholar. No lower publication date limit was imposed in order to capture the earliest relevant contributions to the topic. The review aimed to examine the current understanding of insulin resistance as a medical condition and clinical problem, considering its various classifications.

The database search involved various search terms connected with IR and its management, prioritizing meta-analyses and systematic reviews, research studies considered influential in the fields, as well as the most recently published studies. In order to ensure comprehensiveness and follow the chronology of development of the concept of IR and its classifications, a number of ‘classic’ studies from the mid-to-late 20th century have been included. In total, 86 articles were included in the analysis. The process of selecting and evaluating articles for inclusion in the review generally followed the PRISMA guidelines, insofar as the specific scope of this article allowed. Additionally, the reference lists of the initially selected articles were manually screened to identify further relevant publications.

The MCIR classification of insulin resistance was created by critically evaluating and collating the available information and new insights into a form that would be both comprehensive and clearly presented.

## 3. Results and Discussion

### 3.1. Classification According to Location

The concept that insulin sensitivity differs between tissues became experimentally accessible with the development of the glucose clamp technique by DeFronzo et al. [15]. Although insulin and glucose levels had been measured in earlier studies, determining the extent to which insulin was acting (or not acting) in individual tissues was at that stage impossible. Nevertheless, researchers were able to indicate specific areas of insulin action/resistance, i.e., the liver and peripheral tissues, mainly skeletal muscles.

In the case of peripheral/muscle IR, insulin fails to adequately stimulate glucose uptake and utilization in peripheral tissues [16], which accounts for postprandial hyperglycemia. During examination using the clamp method, this presents as a reduced glucose disposal rate. The transport of glucose into muscles has been shown to depend on regulated glucose transporters, whose expression and translocation are stimulated by insulin [17]. A study conducted by Schulman et al. [18] further demonstrated that the main intercellular defect in muscle IR is impaired glycogen synthesis in muscles resulting from disrupted glucose transport into the cell.

Possible therapeutic targets in muscle IR are based on physical activity—as it is regarded as the main factor that inhibits or reverses the condition. Thus, the main target consists in:

- Achieving improvement in insulin sensitivity despite inefficient insulin signaling [19].

Considering this target, the following intervention can be suggested:

- Performing physical activity to improve glucose uptake—even when insulin signaling is damaged (this is made possible due to the involvement of pathways independent of insulin) [19].

The key characteristic of hepatic insulin resistance is that it does not suppress endogenous glucose production despite high insulin levels, i.e., the liver continues to produce glucose despite hyperinsulinemia. This process is often responsible for fasting hyperglycemia [20], with ‘hepatic insulin resistance’ identified in 1999 as a metabolic phenomenon separate from muscle and tissue-specific IR [21].

Possible therapeutic targets in hepatic IR should:

- Focus beyond insulin modulation (hepatic lipogenesis remains insulin sensitive, may secondarily exacerbate IR, and lead to MASLD (metabolic dysfunction-associated steatotic liver disease nonalcoholic fatty liver disease)—formerly termed nonalcoholic fatty liver disease (NAFLD)—dyslipidemia, and hypertriglyceridemia);
- Aim at controlling de novo lipogenesis [4].

Considering these targets, the following interventions can be suggested:

- Metformin administration (it may improve only one component of hepatic IR, i.e., glucose levels, not solving the lipid and inflammatory issues) [22].

The discovery that led to identifying adipose-tissue IR is the mutual impact of fats and glucose on insulin sensitivity. It was established early on that insulin inhibits lipolysis, i.e., the breakdown of triglycerides into free fatty acids (FFA), in adipose tissue. This effect, however, is known to be weaker in some people with diabetes and obesity, compared to healthy individuals, despite high insulin levels [9]. This discrepancy was explained by Jensen et al. [23], who showed that insulin fails to sufficiently suppress lipolysis, leading to persistently high levels of free fatty acids. In turn, elevated FFA levels may secondarily exacerbate IR in the muscles and the liver through the glucose-fatty acid cycle (the Randle cycle) creating a metabolic vicious circle.

Chronologically speaking, the discovery of peripheral IR predates that of both muscle and hepatic IR. The term ‘peripheral utilization of glucose’, later changed into ‘peripheral IR’, appeared in an article published in 1959 by Goto et al. [24] and denoted the phenomenon whereby glucose uptake in peripheral tissues was not adequate in diabetic patients. The results of the fundamental study conducted by DeFronzo et al. [15] made it possible to distinguish between hepatic and peripheral insulin sensitivity. Using the clamp method, it was shown that the majority of glucose uptake during insulin stimulation occurs in peripheral tissues, mainly in muscles. The term ‘peripheral insulin resistance’ was used in 1983 in a study which showed that despite normal insulin action in the liver, peripheral tissues (mainly muscles) may still not take up glucose efficiently [25]. It was later clarified that ‘peripheral’ IR primarily reflects impaired insulin action in skeletal muscles and adipose tissue resulting from defects in glucose transport (GLUT4), reduced activity of the IRS-PI3K-Akt pathway, intracellular lipid accumulation, or low-grade inflammation [26].

Possible therapeutic targets in adipose-tissue IR should focus on:

- Restoration of normal adipocyte function as an energy buffer—by reducing pathological basal lipolysis and the associated excessive influx of free fatty acids into insulin-sensitive tissues [27].

Possible interventions:

- Performing physical activity—this leads to improved insulin sensitivity, with the effects being more pronounced in those persons whose body weight decreases; this effect, however, is partly independent of weight loss;
- Body weight reduction (as a co-intervention) [28].

**Figure 1:**
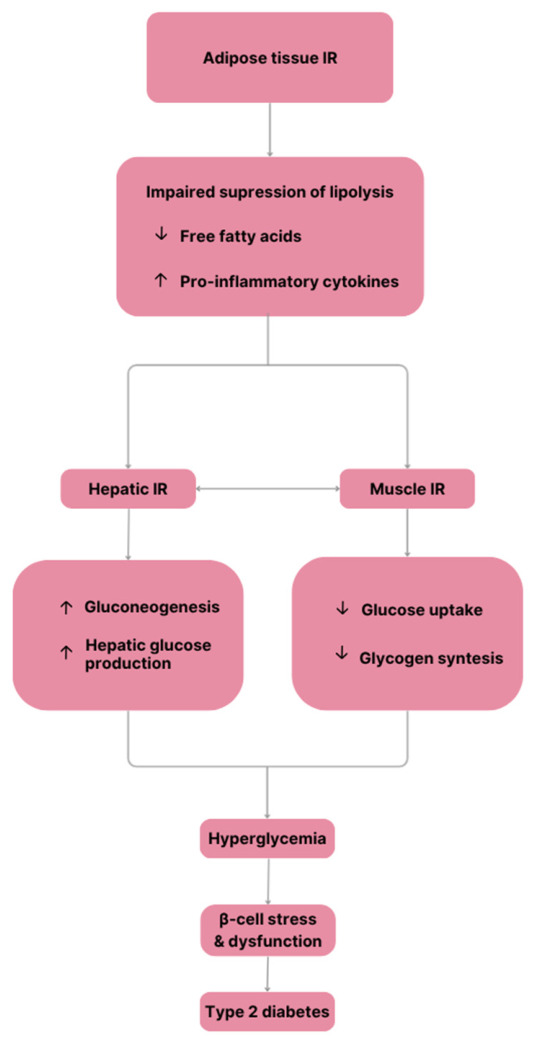
Schematic presentation of the development of insulin resistance. The arrows pointing in one direction represent potential causality, i.e., potential development of IR and its consequences. The arrow pointing in both directions indicates independent cooccurrence of IR types.

Figure 1 presents a schematic illustration of the development of IR, from adipose tissue involvement to the development of type 2 diabetes.

As far as possible therapeutic agents are concerned, the potential role of GLP-1 receptor agonists (GLP-1RAs) needs to be emphasized, which will most probably become useful in IR management. However, these compounds do not act on a single specific type of insulin resistance; rather, they exert pleiotropic effects, influencing several mechanisms associated with IR, including adipose tissue dysfunction, lipotoxicity, chronic inflammation, hepatic insulin resistance, and potentially central insulin resistance. As a result, GLP-1RAs can simultaneously alleviate multiple forms of insulin resistance, in particular by reducing body weight, but also by decreasing ectopic fat accumulation, attenuating inflammatory signaling, and improving metabolic regulation. This is certainly a promising new research direction that must be explored in future studies.

Another type of insulin resistance was proposed in 1978 by Havrankova et al. [29], who demonstrated the existence of insulin receptors in the brain. This meant that although insulin had long been considered a peripheral hormone, it is in fact capable of crossing the blood–brain barrier and that neurons possess insulin receptors. Hence, this discovery provided the basis for further research into how insulin works in the brain and introduced the concept of central insulin resistance. The functional role of insulin in the brain was established in 2004 by Benedict et al. [30], which may have contributed to the concept of ‘brain insulin resistance’ to signify disturbances of neuronal response. In addition, Craft and Watson [31] showed that neurons may also become insulin dysregulated, which became the basis for ‘neuronal insulin resistance’. They also established that insulin modulates neuroplasticity, memory, and appetite and that insulin signaling in the brain is impaired in people with Alzheimer’s disease and obesity. The results of a study performed by Talbot et al. [32] biochemically confirmed the presence of central IR among patients with Alzheimer’s disease. In addition, it was ascertained that central IR is observed regardless of the patient’s diabetes status. A review published by Kullmann et al. [33] demonstrated that central IR is associated with obesity, diabetes, and Alzheimer’s disease. The condition is also linked to impaired insulin transport across the blood–brain barrier and disruptions in IRS-PI3K-Akt signaling in neurons, primarily affecting the hypothalamus (appetite regulation) and the hippocampus (memory and learning). For this reason, insulin levels are taken into account when strategies for combating Alzheimer’s disease are designed.

Possible therapeutic targets in central IR are based on the biological fact that role of insulin in the brain is to inhibit the production of insulin and increase glucose uptake in peripheral tissues. Thus, the main targets consist of:

- Restoration of the brain’s sensitivity to insulin by restoring postprandial energy flow coordination throughout the body [34];
- Slowing the progression of neuropsychiatric disorders (indirect protection of cognitive functions and mood stemming from the association between central IR and Alzheimer’s disease, depression, and schizophrenia) [35],
- Regulation of appetite and response to food stimuli [34].

Considering these targets, interventions should focus on:

- Early treatment of IR, i.e., before the development of advanced neurodegenerative or neuropsychiatric pathology [35].

**Figure 2:**
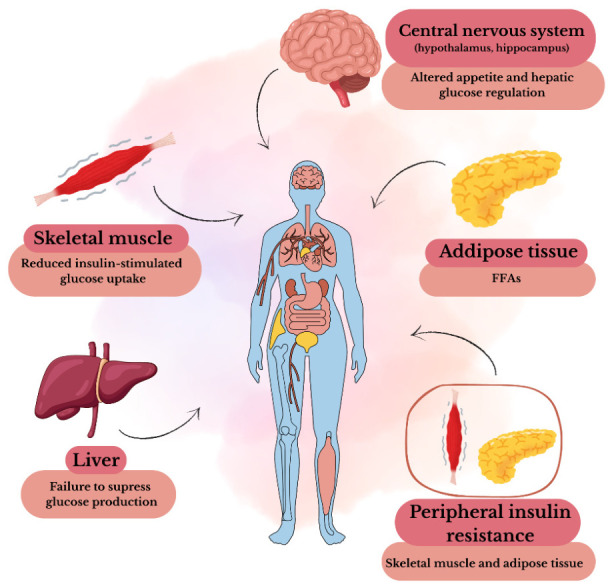
Major anatomical locations of insulin resistance. Central insulin resistance includes insulin resistance affecting brain regions involved in metabolic regulation, including the hypothalamus and hippocampus.

A schematic illustration of location-specific types of insulin resistance is presented in Figure 2.

### 3.2. Classification According to the Molecular Mechanisms

Receptor-level IR was identified based on the observation that patients with obesity, and to a lesser extent those with type 2 diabetes, are characterized by a reduced number of insulin receptors. A study focusing on acquired IR performed by Olefsky and Reaven [36] showed that an impairment in insulin binding to its receptor or a reduced affinity of insulin for its receptor may be present in individuals with obesity and diabetes. Kahn et al. [37] improved on the concept by focusing on syndromes characterized by severe insulin resistance in patients in whom the marked decrease in insulin binding was a primary genetic defect rather than a consequence of hyperinsulinemia. In this manner, the researchers proved that IR may in some cases result from a genetic defect rather than being acquired, e.g., due to obesity. In the proposed MCIR classification (Table 1), the phenomenon is termed ‘receptor insulin resistance’.

An important development of the concept of receptor insulin resistance was made when type B autoimmune insulin resistance (TBIRS) was described. It is characterized by the presence of anti-insulin receptor auto-antibodies, with the condition improving in some patients following immunosuppressive treatment [38]. These findings were further developed by Taylor et al. [39], who also developed the distinction between receptor and post-receptor IR and proposed the classification of IR into types A (genetic/congenital; not autoimmune), i.e., caused by a mutation in the insulin receptor gene or in its function, and B (acquired; autoimmune), i.e., characterized by the presence of antibodies directed against the insulin receptor. This is considered an important moment in the history of molecular diabetology, as it provided strong evidence for the genetic basis of type A insulin resistance and the key role of the INSR gene in insulin action. The clinical phenotypes, e.g., Donohue syndrome or Rabson-Mendenhall syndrome, and mutations of the INSR gene were described in 2002 by Longo et al. [40], who compared the published INSR mutations and connected them with clinical phenotypes, concluding that the more an INSR mutation limits the quantity or activity of the receptor, the more severe the clinical phenotype.

Possible therapeutic targets and interventions in receptor IR should include:

- Metabolic stabilization and ensuring survival as the key therapeutic aim [41];
- Stabilization of blood glucose and reduction in glycemic variability (disease management rather than cure) [42];
- Elimination of the factor blocking the insulin receptor, i.e., insulin receptor autoantibodies (Type B) (unless the receptor blockade is removed, tissues cannot be ‘sensitized’ through diet, exercise, or metformin);
- Reversal of the hypercatabolic state—by administering very high doses of insulin (not ‘treat’ IR but rather ‘save’ the metabolism) [43];
- Bypassing the defective insulin receptor signaling (bypass signaling) [41];
- Bypassing the defect in an insulin receptor, i.e., signaling through insulin-independent glucose-lowering mechanisms (metabolic bypass);
- Prevention and monitoring of hypoglycemia and normoglycemic ketoacidosis [42].

Due to the very low prevalence of receptor-related insulin resistance (IR), intervention research is limited mainly to case studies, with a marked lack of large cohort studies. The possible interventions could include:

- Continuous administration of recombinant IGF-1 (rhIGF-1) using an insulin pump (Donohue syndrome) [44];
- Use of empagliflozin (an SGLT2 inhibitor) [45] or long-term treatment with metreleptin [46] (Rabson–Mendenhall (RM) syndrome).

A comprehensive description of the mechanism of action of the insulin receptor from the molecular level to the metabolic effect was described by Kahn et al. [47], while Nishimura et al. [48] provided evidence that because the insulin receptor appears to function normally in many subjects with IR, the defect must lie in the intracellular processes that occur after insulin binds to its receptor. Post-receptor defects in insulin action have been demonstrated to constitute the dominant mechanism of IR in common metabolic diseases, with the accumulation of lipids in muscle and liver tissue identified as their main cause. Lipid accumulation occurs when excess fatty acids in muscle lead to the formation of lipotoxic metabolites, e.g., ceramides, activating serine-threonine kinases that phosphorylate IRS-1/IRS-2 and block insulin’s ability to activate the PI3K-Akt pathway. This results in impaired GLUT4-mediated glucose transport. Unlike the Randle cycle, this mechanism shows that intermediate lipid metabolites, rather than triglycerides, directly impair insulin action upstream of glucose metabolism [49]. Thus, IR does not always stem from abnormalities of the receptor, as intracellular (post-receptor) mechanisms are a significant—and often predominant—factor in, e.g., obesity or type 2 diabetes. The term ‘post-receptor defect’ was therefore used in a purely functional sense, while the biological development of the molecular model of insulin signaling was presented in 1992, when a modern model of the insulin signaling pathway based on the tyrosine-kinase receptor and insulin receptor substrates (IRS) was introduced [50]. The model also showed that metabolic IR is not equivalent to a complete loss of insulin signaling; it is instead associated with selective weakening of the PI3K/AKT metabolic pathway. The modern molecular model of insulin action integrates glucose and lipid regulation and makes it possible to identify precisely defined and localized sources of post-receptor IR within the IR-IRS-PI3K-Akt-GLUT4 signaling pathway, as well as in the mechanisms governing lipid metabolism [51].

Hence, at the molecular level, numerous mechanisms can contribute to impaired insulin signaling. Under physiological conditions, the binding of insulin to the insulin receptor activates IRS-1 and IRS-2 proteins and downstream PI3K/AKT signaling, leading to glucose uptake and metabolic regulation. In IR, this pathway can be disrupted at several levels. Chronic inflammation activates pathways dependent on TNF-α, JNK, and NF-κB, resulting in inhibitory serine phosphorylation of IRS-1 and IRS-2 proteins. Excess lipid accumulation, on the other hand, promotes the formation of diacylglycerols, which activate protein kinase C isoforms and impair insulin signaling. Consequently, IRS-1 and IRS-2 proteins, PI3K/AKT signaling, JNK, PKC, NF-κB, and related molecules represent key nodes linking different mechanistic forms of insulin resistance.

Inflammatory processes are also associated with IR. Hotamisligil [52] showed that low-grade inflammation in adipose tissue, the liver, or muscle leads to post-receptor defects in insulin signaling through the activation of inflammatory kinases. The concept of Inflammatory IR was thus seen as a form of post-receptor IR. This concept is an important link between the inflammatory aspects of obesity and the molecular mechanisms underlying metabolic IR. All the main mechanisms of post-receptor IR, i.e., lipid, inflammatory, mitochondrial, and stress-related, were presented as a single coherent pathophysiological model in 2012 by Samuel and Shulman [53].

The overarching therapeutic target in post-receptor IR is increasing GLUT4 translocation [54] through:

- Restoration of proper IRS function;
- Increasing the availability and function of GLUT4 [55].

Other possible therapeutic targets could involve:

- Restoration of an efficient PI3K-Akt axis;
- Reduction of chronic activation of mTORC1/S6K1 [55];
- Activation of AMPK as a compensatory pathway (AMPK bypasses impaired insulin signaling pathway and restores glucose uptake independently of IRS/PI3K) [54,55].

Considering these targets, interventions could include:

- Reduction of oxidative stress and inflammation that impair IRS signaling [54];
- Use of metformin, which activates AMPK and reduces hepatic glucose production.

Another type of IR is transport insulin resistance, in which insulin receptor signaling is active but GLUTs are not properly translocated to the cell membrane or activated [56]. This phenomenon results from the impairment of the translocation and function of glucose transporters (GLUT) and is considered a downstream manifestation of post-receptor insulin resistance [57]. As mentioned earlier, the main insulin-sensitive glucose transporter responsible for insulin-stimulated glucose uptake into skeletal muscle and adipose tissue is GLUT4 [58]. The molecular and cellular basis of insulin-sensitive glucose transport, combining earlier biochemical data with new knowledge on the structure, expression, and regulation of GLUT4, was presented by Birnbaum [59]. He also emphasized that GLUT4 is the major insulin-responsive glucose transporter, whose activity depends on the intracellular redistribution of the protein rather than changes in gene expression. Additionally, he proposed that GLUT4 expression could serve as a biomarker of insulin sensitivity. Insulin acts through a complex system of membrane and vesicular microdomains that control GLUT4 translocation [60], with the protein AS160 (AS160/TBC1D4) identified as a key effector of the kinase AKT2, responsible for GLUT4 vesicle translocation to the plasma membrane, which acts as a brake on GLUT4 translocation [61].

Possible therapeutic targets in transport IR could involve:

- Restoration of insulin-stimulated GLUT4 translocation;
- Reduction of mitochondrial oxidative stress (mitoROS) [62];
- Bypassing the defect in insulin signaling through the activation of energy-sensing pathways [19].

Considering these targets, interventions could focus on:

- Performing physical activity—regarded as a structural intervention rather than a ‘blood glucose-lowering agent’. In this context, insulin and exercise must be convergent at the level of TBC1D1/TBC1D4 [19].

The concept of mitochondrial insulin resistance was proposed by Kelley et al. [63] based on direct histological and biochemical evidence confirming that muscle IR has a mitochondrial basis. It had earlier been discovered that the main intracellular metabolic defect in IR is impaired glycogen synthesis in skeletal muscles [18]. In humans with type 2 diabetes, structural and functional mitochondrial dysfunction in skeletal muscles was found to be associated with the severity of IR [63]. However, a deficit in mitochondrial function and a decrease in mitochondria size observed with aging is also observed in the muscles of healthy older individuals, regardless of the presence of obesity or diabetes. This observation led to the conclusion that aging is associated with the onset of IR through mitochondrial energetic disturbances [64]. It was later observed that mitochondrial dysfunction is indeed present not only in type 2 diabetic patients, but also in healthy older individuals [65]. The strongest evidence for the existence of primary, hereditary mitochondrial IR was provided by Petersen et al. [66], who demonstrated that mitochondrial dysfunction and IR can be present in young, lean individuals genetically predisposed to type 2 diabetes before the onset of obesity or hyperglycemia. In other words, IR can develop under ‘normal’ metabolic conditions if mitochondria have a reduced capacity for fatty acid oxidation. The role of mitochondria in IR was later found to be more complex, with a dysregulation not always implying a ‘deficit’ [67]. The concept introduced and developed by Shulman et al. [18,65] was later expanded with the notion of metabolic flexibility defined as the ability of muscle to switch rapidly between fatty acid oxidation and glucose utilization.

Possible therapeutic targets in mitochondrial IR could focus on:

- Activation of AMPK as a central therapeutic target [68];
- Reduction of mitochondrial ROS as a prerequisite for restoring glucose transport;
- Improvement of mitochondrial oxidation to remove the lipid blockade of glucose transport [69];
- Reduction of pathological hepatic glucose production (HGP) [68].

Considering these targets, interventions could focus on:

- Performing physical activity—to simultaneously improve mitochondrial function and enable GLUT4 transport [69];
- Reduction of oxidative and endoplasmic reticulum (ER) stress as secondary amplifiers of IR [54].

The molecular model of lipid interference was first formulated as a mechanism in which the accumulation of lipids in muscle and liver indirectly blocks insulin signaling through the activation of PKC protein kinases [70]. It was then shown that muscle IR is not only a signaling (IRS-AKT) problem but also involves metabolic disturbances characterized by the mitochondria’s loss of ability to switch between fatty acid oxidation and glucose utilization [71]. Clinical in vivo evidence in humans pointing to lipid-induced insulin resistance was presented by Itani et al. [70], who showed that the accumulation of diacylglycerol (DAG) in muscle is indeed associated with the activation of PKCθ and leads to IR. Although an integrated model of IR—combining lipid, mitochondrial, inflammatory, and hormonal pathways into a single coherent pathophysiological framework—was later proposed [53], other processes such as oxidative stress, inflammation, and endoplasmic reticulum stress, as well as the microbiota, were also found to modulate this central mechanism. Zhang et al. [72] indicated that the key factor in lipid-induced IR is not the excess of lipids itself, but rather the dysregulation of lipid-derived signaling molecules and nutrient signaling pathways.

Possible therapeutic targets in lipid-induced IR could involve:

- Reduction of pathological HGP to restore insulin control of gluconeogenesis instead of merely reducing peripheral blood glucose;
- Disruption of the DAG-PKCε-IRS-PI3K axis to reduce DAG signaling instead of merely ‘stimulating’ insulin production;
- Activation of AMPK as a therapeutic hub to simultaneously improve lipid metabolism and hepatic insulin sensitivity;
- Reduction of lipid-induced IR in hepatocytes to decrease lipid overload, as it initiates hepatic IR [68].

Considering these targets, interventions could focus on:

- Dietary interventions aimed at reducing body weight [73].

As far as inflammatory insulin resistance is concerned, overexpression of inflammatory factor TNF-α (tumor necrosis factor alpha) in the adipose tissue of obese individuals has been found to directly induce IR [74]. The discovery of TNF-α as a mechanism potentially inducing IR was then linked to a specific signaling mechanism, identifying the c-Jun N-terminal kinase (JNK) as the key mediator of metabolic inflammation leading to IR [75]. Weisberg et al. [76] expanded the concept of inflammatory IR by including immunological aspects, demonstrating that obesity is associated with macrophage infiltration of adipose tissue by macrophages, which secrete pro-inflammatory cytokines. The third key aspect was introduced by Özcan et al. [77] in the form of ER stress as a molecular link between obesity, inflammation, and impaired insulin signaling. In obesity, cells experience metabolic overload, leading to the activation of ER stress, which in turn triggers the same stress kinases as pro-inflammatory cytokines. The innate immune system responds to an excess of fatty acids, triggering a mechanism that leads to combined lipid-inflammatory IR [52,78]. Metabolic inflammation was further linked to the digestive system—more precisely, the gut microbiota—with a slight increase in blood levels of bacterial endotoxins (LPS) originating from the gut shown to trigger chronic inflammation and IR. The underlying premise is that a high-fat diet raises blood LPS levels, which in turn activates the TLR4 pathway, initiating low-grade chronic inflammation that ultimately leads to obesity-related IR [79]. The concept of inflammatory IR was then extended to involve the brain, particularly the hypothalamus (central inflammatory IR). Thaler et al. [80] showed that obesity leads to inflammation, and neuronal damage in the arcuate nucleus (ARC) of the hypothalamus, a region responsible for regulating energy homeostasis.

Possible therapeutic targets in inflammatory IR could involve:

- Neutralization of TNF-α overexpressed in adipose tissue in obesity;
- Inhibition of TNF-α production and signaling [81];
- Reduction of TNF-α, a pro-inflammatory cytokine [82] (this is uncertain: the literature data concerning the role of TNF-α is contradictory in conclusions [83]);
- Neutralization of TNF-α overexpressed in adipose tissue in obesity; inhibition of TNF-α production [81]; reduction of TNF-α, a pro-inflammatory cytokine (potentially) [82]; however, literature data concerning the role of TNF-α is contradictory in conclusions [83];
- Inhibition of JNK activity to restore IRS-1 functionality and improve insulin sensitivity without increasing insulin levels;
- Instead of ‘stimulating IRS-1′: (1) removing inhibitors of IRS-1 activity, (2) restoring the predominance of tyrosine phosphorylation over inhibitory serine phosphorylation [83].

Considering these targets, interventions could focus on:

- Dietary interventions aimed at reducing body weight and inflammation [84].

**Figure 3:**
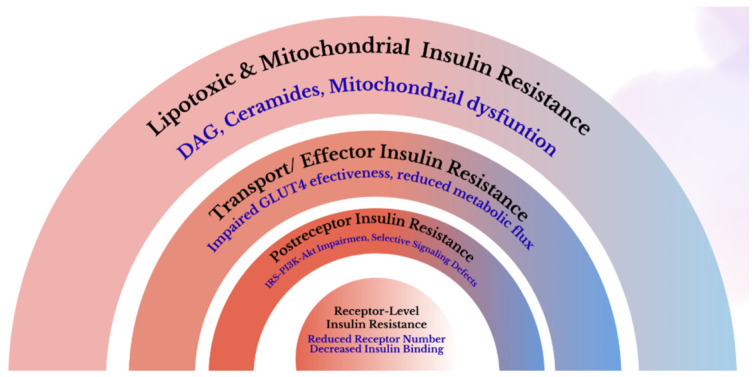
Presentation of insulin resistance according to molecular mechanisms.

Figure 3 illustrates the different types of insulin resistance based on their underlying mechanisms. The different stages of insulin signaling are presented as the respective layers and indicate the specific mechanisms that cause IR.

### 3.3. Classification According to Clinical Cause

Although in rare cases IR is a primary condition caused by genetic mutations that affect the function of the insulin receptor or post-receptor signaling pathways, in most cases IR is acquired. In the case of physiological IR, key evidence for the functional significance of the condition was first identified in a study performed in a group of pregnant women with the description of the concept of accelerated fasting [85] and confirmed when pregnant women were examined longitudinally using the clamp technique to measure insulin sensitivity and insulin secretion. It was thus demonstrated that a physiological decrease in insulin sensitivity of about 50–60% occurs during pregnancy, which is compensated by an increase in insulin secretion [86]. A similar phenomenon was identified in the case of puberty, when insulin sensitivity is lower by about 30% during puberty—associated with increased human growth hormone (hGH) secretion—and returns to prepubertal levels after the growth period ends [87]. A similar situation has been observed in critically ill patients in whom the intensive insulin therapy was administered to maintain normoglycemia, which was associated with reduced mortality and the risk of infection [88]. Thyfault and Booth [89] introduced the concept of adaptive IR resulting from decreased physical activity, which may occur in persons who are healthy and do not experience changes in body weight. Interestingly, the condition is not considered pathological when it is short-term (physiological-adaptive) and occurs when skeletal muscles stop working regularly, e.g., due to immobilization, prolonged sitting, lack of exercise, or during periods of reduced muscle activity. In such cases, when this state becomes prolonged, it turns into a pathological factor contributing to the development of diabetes and metabolic syndrome.

Possible therapeutic targets in adaptive IR could involve:

- Activation of AMPK;
- Improvement of skeletal muscle insulin sensitivity;
- Restoration of AMPK–mTOR signaling balance;
- Improvement of mitochondrial function and reduction in lipotoxicity [90].

In the case of adaptive IR, specific interventions depend on the underlying clinical cause rather than addressing IR as the target condition.

### 3.4. Classification According to Metabolic Context

The main phenomenon connected with IR in the metabolic context is metabolic obesity in individuals with normal body weight. Metabolic obesity is associated with insulin resistance manifesting through increased visceral adiposity despite normal body weight, showing that IR can occur independently of overall obesity [91]. Thus, obesity is not a necessary pre-condition for the development of IR as genetics, fat distribution, and phenotypic traits can determine IR independently of body weight. In this respect, lean IR is identified as primarily visceral fat accumulation and ectopic lipid deposition in metabolic tissues [92]. This condition, termed ‘lean metabolic syndrome’, refers to metabolic syndrome and IR in individuals with normal BMI [93].

Possible therapeutic targets in lean IR could focus on:

- Reduction of adiposity and improvement of body composition;
- Reduction of hepatic fat accumulation [94].

Considering these targets, interventions could focus on:

- Lifestyle modifications as the cornerstone of therapy [94].

As mentioned earlier, IR is considered the central pathophysiological mechanism underlying various metabolic and cardiovascular conditions, which are mainly lifestyle-related [1,95]. With this underlying principle in mind, obesity-related IR was further explained with the discovery of inflammation in adipose tissue, particularly the overexpression of TNF-α, which plays a direct role in the development of the condition [74]. The latest review of the current understanding of the mechanisms of obesity-related IR was performed in 2017 by Engin et al. [96], who focused on the molecular mechanisms of adipose tissue inflammation in obesity leading to IR. They indicate that adipocytes are conducive to the initiation of the inflammatory signal through their production of cytokines and chemokines. The main molecular inflammatory pathways leading to IR include TLR4, activated by saturated fatty acids and producing cytokines; JNK, which inhibits insulin signaling; NF-κB involved in the transcription of inflammatory genes; and ER stress activating JNK and worsening insulin signaling. In obesity-related IR, ceramides, ROS, mitochondrial damage, and excess free fatty acids activate the NLRP3 inflammasome-caspase-1-IL-1β pathway. Importantly, adipose tissue hypoxia, resulting from enlargement of adipocytes and insufficient vascularization, leads to oxygen deprivation, activating HIF-1α and impairing adipose tissue function.

Possible therapeutic targets in obesity-related IR could focus on:

- Achieving a certain percentage of body weight reduction that would lead to improvement in various metabolic disturbances (even a 2–5% body weight loss improves glucose levels and IR, while a body weight loss of 5–10% produces clinically meaningful improvement in IR and prevents type 2 diabetes; further weight loss can lead to additional benefits (particularly for muscle IR), though this may only apply up to a certain threshold) [97];
- Reduction of pathological lipid intermediates (DAG, ceramides) in the liver and muscle to restore insulin signaling [53].

Considering these targets, interventions could focus on:

- Lifestyle modifications and physical exercise to achieve body weight reduction;
- Suppression of low-grade inflammation that drives IR in obesity [52].

Stress-induced insulin resistance is rooted in the concept of stress as a systemic biological response to harmful stimuli and described by Selye [98] in his theory of the general adaptation syndrome. Exton [99] described how stress hormones (glucagon, adrenaline, and cortisol) and insulin regulate hepatic glucose metabolism. Stress hormones were shown to increase glucose production, with insulin acting antagonistically. Van den Benghe et al. [88] demonstrated the clinical significance of stress-induced (acquired) IR in critically ill patients with a reduction in ICU mortality of over 40%. The idea that intensive insulin therapy can dramatically improve outcomes was thus introduced with stress hyperglycemia described as a manifestation of IR triggered by metabolic, hormonal, and inflammatory stress. It has also been demonstrated that chronic psychological stress induces low-grade inflammation through the activation of the HPA axis and cytokine production, which may in turn disrupt insulin signaling (IRS-1 PI3K/AKT), contributing to the development of psycho-inflammatory IR [100,101].

Possible therapeutic targets in stress-induced IR could involve:

- Regulation of cortisol secretion and normalization of HPA axis activity;
- Restoration of glucocorticoid receptor (GR) and insulin receptor sensitivity;
- Suppression of low-grade, chronic inflammation (linking stress and IR) through reduction of TNF-α, IL-6, CRP, and inhibition of JNK/NF-κB signaling [102].

Considering these targets, interventions could focus on:

- Normalization of the circadian rhythm (links between sleep disturbances and IR) [102].

On the basis of the above analysis of the various types of IR and their possible therapeutic targets and interventions, the Multidimensional Classification of Insulin Resistance (MCIR) was created. The IR types marked with an asterisk (*) are new ones proposed by the authors of this study. Out of all the IR types discussed above, adaptive and lean IR were not included in the MCIR classification—a decision was made to subdivide those into other categories. It should be noted that the proposed categories are not mutually exclusive and may overlap across multiple physiological and clinical dimensions, as IR is a multidimensional phenomenon and different mechanisms may coexist and interact within the same individual. Thus, the proposed categories are intended to represent dominant pathophysiological mechanisms or levels of biological organization rather than mutually exclusive clinical entities. The MCIR classification is presented in Table 1.

**Figure 4:**
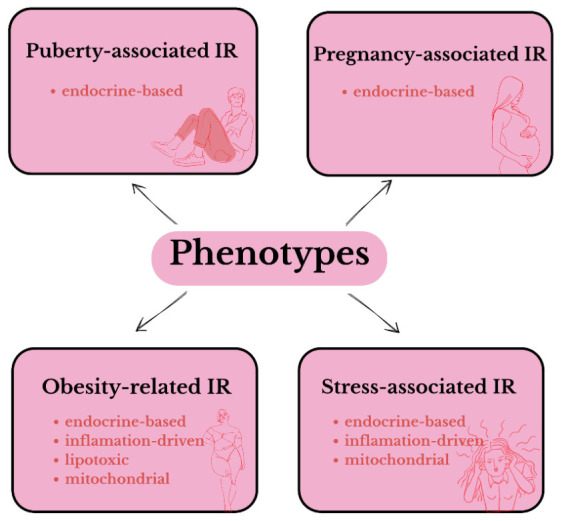
Phenotypes of insulin resistance and their respective underlying mechanistic categories.

Although IR presents as a heterogeneous and multidimensional condition, several core therapeutic principles are broadly applicable across its subtypes and remain fundamental to reducing the risk of serious complications. These interventions target shared pathophysiological pathways such as excess adiposity, ectopic fat accumulation, chronic low-grade inflammation, and metabolic dysregulation (Figure 4). Hence, key universal strategies include sustained weight management, regular physical activity, dietary modification, improvement of sleep quality, stress reduction, and comprehensive management of cardiometabolic risk factors such as dyslipidemia, hypertension, and impaired glucose regulation. GLP-1RAs could also be tentatively added to the list; it needs to be clarified, however, that they do not target IR specifically or a particular type of IR. While the relative importance and implementation of particular interventions may vary depending on the dominant mechanistic subtype of insulin resistance, the approaches specified in the MCIR classification collectively represent the foundational approach to prevention and long-term metabolic health.

The relations between phenotypes of IR and their underlying mechanistic categories presented above can thus be regarded as the central conceptual framework of universal insulin resistance therapies, from which individual intervention types can be designed to support specific physiological targets. Such a structure emphasizes that despite mechanistic diversity, there is a shared therapeutic core that applies across the whole IR spectrum, which makes it possible to protect the patient against downstream complications.

## 4. Conclusions

Insulin resistance is an exceptionally heterogeneous condition, which is why its classification and clinical implications are complex and problematic issues. IR can be either congenital or acquired with some individuals predisposed to it more than others. Taking this into consideration, the authors’ proposed novel Multidimensional Classification of Insulin Resistance (MCIR) is envisioned as a comprehensive, ordered grouping of the various types of the condition. Moreover, the tabular and graphic presentation is aimed at possible clinical applications, to facilitate both the understanding of IR and making accurate clinical and therapeutic decisions. In addition, it needs to be emphasized that further research in the area of insulin resistance needs to focus on the interdisciplinary nature of the condition. The involvement of experts in clinical medicine, dieticians, endocrinologists, family doctors, and other professionals such as personal trainers would make it possible to better adapt the considerable existing knowledge base to the varying needs of individual patients. Another important research direction would be to characterize insulin signaling and related pathways (e.g., TNF-α, JNK, LPS/TLR4, and DAG signaling) and identify key molecular nodes—including second messengers, kinases, phosphatases, and downstream effectors—whose dysregulation contributes to IR. In view of the clinical importance of insulin resistance, the proposed comprehensive MCIR classification that both summarizes and expands upon the existing state of IR research may prove very useful both as a clinical guideline and a signpost for future research directions.

## 5. Limitations of the Study

The main limitation connected with IR research in general is the large number of proposed types of IR that have appeared in the literature. In addition, diagnostics of the condition are made difficult either due to the limitations inherent in the methods used to assess insulin resistance (HOMA-IR, the Matsuda index) or insufficient availability (the clamp). This makes it difficult to classify the various types of IR. Another issue stems from the fact that insulin resistance is a condition whose history is characterized by the emergence of numerous—often independent—research directions. As a result, the literature data contain certain ideas and approaches that are often incompatible or difficult to consider as part of the established scientific consensus. For this reason, the authors decided to omit ‘fringe’ ideas from the review and the MCIR classification if they considered them insufficiently tested, speculative, and/or inconsistent with the current scientific consensus.

## Figures and Tables

**Table 1:** The Multidimensional Classification of Insulin Resistance (MCIR) and the respective therapeutic aims. Asterisk (*) denotes IR types proposed by the authors of this study.

General Category	Category of IR	Nature of IR (Physiological, Pathological, or Context-Dependent)	Typical Context	Therapeutic Interventions
Tissue-specific IR	Muscle IR	context-dependent	skeletal muscle IR in obesity/exercise	performing physical activity to improve glucose uptake
	Hepatic IR	context-dependent	MASLD, fasting, T2D	(1) control of de novo lipogenesis (2) metformin administration
	Adipose-tissue IR	context-dependent	obesity, lipolysis dysregulation	restoration of normal adipocyte function
	Central IR	context-dependent	obesity, neurodegenerative disorders	(1) regulation of appetite and response to food stimuli (2) early IR treatment, before the development of pathology
Molecular/mechanistic IR	Receptor IR	pathological	rare, severe genetic syndromes (Type A/B IR)	Interventions focused on IR management: (1) metabolic stabilization (2) stabilization of glucose levels (3) reversal of the hypercatabolic state by administering high doses of insulin
	Post-receptor IR	mostly pathological	T2D, obesity	(1) administration of metformin (2) reduction in oxidative stress and inflammation
	Transport IR	mostly pathological	impaired GLUT4 (T2D, inactivity)	performing physical activity
	Mitochondrial IR	pathological (potentially reversible)	aging, inactivity	(1) performing physical activity (2) reduction in oxidative and ER stress
	Lipid-induced IR	pathological	obesity, ectopic fat, MASLD	dietary interventions aimed at reducing body weight
	Inflammatory IR	pathological	obesity, chronic inflammation	dietary interventions aimed at reducing body weight and inflammation
Endocrine/context-related IR	Pregnancy-associated IR *	physiological (may become pathological (GDM))	pregnancy	may progress to gestational diabetes mellitus (GDM) if β-cell compression fails; requires monitoring and glycemic control
	Puberty-associated IR *	physiological (transient)	adolescence	transient, no intervention required; may unmask metabolic risk
	Obesity-related IR	pathological	obesity, metabolic syndrome	(1) weight loss strategies, including diet and physical activity (2) suppression of low-grade inflammation
	Stress-induced IR	physiological (acute)/pathological (chronic or severe)	stress	(1) acute glycemic management may be required in critical illness (2) normalization of the circadian rhythm/sleep patterns
	Circadian rhythm-related IR *	context-dependent (physiological/pathological if disrupted)	shift work, circadian disruption	modification of work patterns (e.g., implementing single-shift approaches)
	Sleep deprivation-related IR *	pathological (reversible)	chronic sleep deprivation	normalization of sleep patterns
Physiological/phenotypic	Fasting-induced IR *	physiological	fasting, caloric restriction	adaptive, no intervention required
	Age-related IR *	context-dependent (physiological aging vs. pathological)	sarcopenia, aging, reduced physical activity	lifestyle modifications
	Exercise-induced IR *	physiological (transient, adaptive)	acute physical activity	transient (reflects metabolic adaptation), no intervention required
	Lean phenotype IR *	pathological	Thin Outside, Fat Inside (TOFI), Metabolically Obese Normal Weight (MONW) phenotype	commonly underdiagnosed; requires metabolic assessment despite normal BMI

## Data Availability

No new data were created or analyzed in this study. Data sharing is not applicable to this article.
